# Changes in the characteristics and outcomes of high-risk pregnant women who delivered prior to and after China’s universal two-child policy: a real-world retrospective study, 2010–2021

**DOI:** 10.1186/s12889-024-17810-9

**Published:** 2024-01-31

**Authors:** Caixia Zhu, Shaofeng Zhang, Lixia Shen, Lisha Ye, Minjin Zhan, Shiqin Cai, Jingwan Huang, Zilian Wang, Haitian Chen

**Affiliations:** Department of Obstetrics and Gynecology, The First Affiliated Hospital of Sun Yat-sen University, Guangzhou, China

**Keywords:** Family planning policy, Real-time, Obstetric outcomes, Single pregnancy

## Abstract

**Background:**

In 2016, the “universal two-child” policy, allowing each couple to have two children, was introduced in China. The characteristic change of the long-term period after the implementation of the universal two-child policy was unclear. We studied trends in the obstetric characteristics and their potential impact on the rates of cesarean section and preterm birth in the era of China’s universal two-child policy.

**Methods:**

A tertiary center-based study (2010–2021) retrospectively focused single high-risk pregnancies who delivered from the one-child policy period (OCP, 2010–2015) to the universal two-child policy period (TCP, 2016–2021). A total of 39, 016 pregnancies were enrolled. Maternal demographics, complications, delivery mode and obstetric outcomes were analyzed. Furthermore, logistic regression analysis was used to explore the association between the cesarean section rate, preterm birth and implementation of the universal two-child policy, adjusting maternal age, parity, and fetal distress.

**Results:**

Ultimately a total of 39,016 pregnant women met the criteria and were included in this analysis. The proportion of women with advanced maternal age (AMA) increased from 14.6% in the OCP to 31.6% in the TCP. The number of multiparous women increased 2-fold in the TCP. In addition, the overall rate of cesarean section significantly decreased over the policy change, regardless of maternal age, whereas the risk of preterm birth significantly increased in the TCP. Adjusting for maternal age, parity and fetal distress, the universal two-child policy showed a significantly favorable impact on the cesarean section rate (RR 0.745, 95%CI (0.714–0.777), *P* < 0.001). Compared to the OCP group, a higher increase in fetal distress and premature rupture of membranes (PROM) were observed in the TCP group. In pregnancies with AMA, there was no increase in the risk of postpartum hemorrhage, whereas more women who younger than 35 years old suffered from postpartum hemorrhage in TCP. The logistic regression model showed that the universal two-child policy was positively associated with the risk of postpartum hemorrhage (RR: 1.135, 95%CI: 1.025–1.257, *P* = 0.015).

**Conclusions:**

After the implementation of the universal two-child policy in China, the rate of the cesarean section significantly decreased, especially for women under 35 years old. However, the overall risk of postpartum hemorrhage increased in women under 35 years old, while there was no change in women with AMA. Under the new population policy, the prevention of postpartum hemorrhage in the young women should not be neglected.

## Background

In 1979, the “one-child” family planning policy was implemented to balance the increase in population in China. However, subsequently, the Chinese population exhibited population aging and a decline in fertility, leading to the loss of the previous population excess. Therefore, a “universal two-child” policy was implemented in 2016, allowing each couple to have two children. This two-child policy aimed to increase the population and alleviate the economic problems caused by the aging workforce [1]. With the universal two-child policy, the number of pregnancies at advanced maternal age (AMA, defined as ≥ 35 years old) increased, leading to inevitable obstetric challenges [2]. It has been demonstrated that AMA is associated with complications and adverse pregnancy outcomes, including gestational diabetes mellitus (GDM), preeclampsia (PE), preterm birth, and postpartum hemorrhage (PPH) [3, 4]. Furthermore, a systematic review reported that AMA increased the risk of cesarean Sect. [5]. In China, the proportion of AMA increased to 31% in 2016 [6]. There is no doubt that obstetrics have advanced over the past decade, which might have improved the pregnancy outcomes [1]. Whether the distinct disadvantage from pregnancies at AMA could be compensated by the advantages originating from the medical advancement and high social conomic status still needs to be investigated.

In China, however, long-term studies focused on comprehensive changes after the implementation of the universal two-child policy are far less reported. It is urgent to explore the influence of the universal two-child policy on obstetric changes, which might provide insight into the potential effects of the three-child policy implemented in 2021. Therefore, we aimed to investigate the change of characteristics and outcomes in the context of the universal two-child policy and to further assess the health policies after the introduction of a three-child policy.

In this study, we performed a retrospective cohort survey in a tertiary medical center to identify the maternal and neonatal outcomes under different family planning policies. The potential obstetric changes, associated with family planning policies were assessed to identify specific recommendations to overcome the challenges of the universal two-child policy.

## Materials and methods

### Study design

This is a retrospective study that analyzed the maternal characteristics and obstetric outcomes of pregnancies from 2010 to 2021 in women admitted to the First Affiliated Hospital, Sun Yat-sen University in Guangzhou, China. More than 4,000 pregnant women delivered in our hospital each year and most of cases were high-risk pregnancies. All treatment procedures and health care were in accordance to the approved guidelines and relative regulations. Single pregnancies aged ≥ 18 years old were eligible for in this study. The exclusion criteria included twin pregnancies, multiple pregnancies, and women younger than 18 years old. The present study enrolled 39,016 cases. Among 39,016 pregnant women met these criteria, no cases decided. The Ethics Committee of the First Affiliated Hospital, Sun Yat-sen University approved this research.

To investigate the impact of implementing the two-child policy on the maternal and neonatal outcomes, we divided the past decade into two stages. Owing to the implementation of universal two-child policy in 2016, the period of 2010–2021 was categorized into 2 stages: the one-child policy period (OCP, reference category) and the two-child policy period (TCP). Maternal characteristics included age, parity, obstetric complications, and delivery mode. The neonatal outcomes included gestational age at delivery, stillbirth, birth weight, 1-min, 5-min APGAR scores, neonatal sex, neonatal asphyxia and neonatal death. Delivery mode was grouped into the following categories: vaginal delivery, assisted vaginal delivery with forceps or cesarean section. Advanced maternal age (AMA) was defined as age ≥ 35 years at the expected date of confinement. A woman who has previously given birth is multiparous and the woman who has not given birth is nulliparous. Neonatal asphyxia was defined as 1-min APGAR score ≤ 7 after birth. Preterm birth was defined as birth at < 37 weeks of gestation. Stillbirth was defined as fetal death occurring at ≥ 28 weeks of gestation or during labor, while neonatal death was defined as deaths among live births during the first 28 completed days of life. The obstetric complications include postpartum hemorrhage, fetal distress and premature rupture of membranes (PROM). Postpartum hemorrhage was defined as blood loss of more than 500 ml within 24 after vaginal delivery or 1000 ml after cesarean section. Fetal distress was defined as the condition during pregnancy or labor where the fetus showed signs of inadequate oxygenation. Premature rupture of membranes, previous known as premature rupture of membranes was defined as an amniotic sac break before the onset of labor.

### Statistical analysis

All the data were analyzed by using SPSS software (SPSS version 20.0; SPSS Inc, Chicago, IL). Categorical data are expressed as percentages, while quantitative data are expressed as the mean ± standard deviation (SD). The changes in maternal characteristics and obstetric outcomes over time were compared by the chi-squared test, Fisher’s exact test or Student’s t test. The mean maternal age and mean neonatal birth weight were compared across past decade by Student’s t test, while the chi-squared test was used to compare the risk of AMA across the past decade. The association between the two-child policy and the risks of cesarean section and postpartum hemorrhage were estimated by logistic binomial regression models, analyzing adjusted relative risks (RRs) with 95% confidence intervals (CIs). Data from the OCP were regarded as the reference group, while the date from the TCP was considered the comparison group. The potential covariates included parity, maternal age and fetal distress. *P* < 0.05 was considered statistically significant.

## Results

### Demographic characteristics

**Fig. 1:**
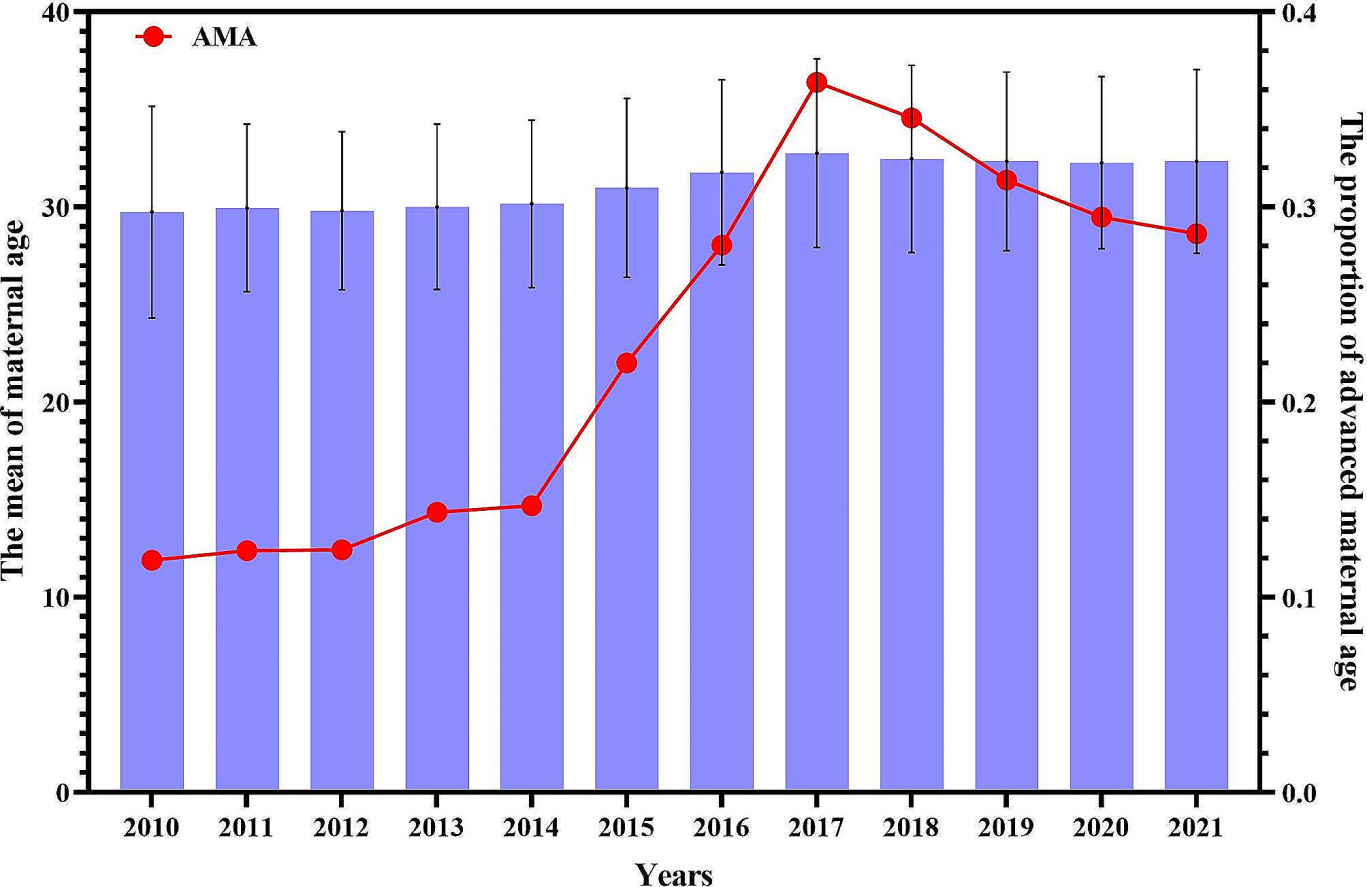
The maternal age at delivery over years before and after the universal two-child policy. *AMA: advanced maternal age

Ultimately a total of 39 016 single pregnancies that gave birth were included in this analysis. Owing to the surveillance data, the number of pregnancies had increased to 20,601 during the TCP period, reflecting an 11.8% increase over the OCP period (*N* = 18,415). The mean maternal age at pregnancy was 30.0 ± 4.46 years in the OCP period, while the mean age was 32.3 ± 4.68 years in the TCP period with a significant difference over time. Furthermore, the annual mean age was generated as a line plot for each year from 2010 to 2021 (Fig. 1). Maternal age increased after the implementation of the universal two-child policy, with a peak in 2017. As shown in Table 1, the proportion of women with AMA was 14.5% in the OCP period, whereas this proportion was 31.6% during the 5-year TCP period. Figure 1 depicts the increasing trend in the overall proportion of women with AMA ffrom the OCP period to the TCP period, which peaked in 2017. The rate of multiparity steadily increased after the policy change, from 25.4 to 47.6%.

**Table 1:** The demographic characteristics and obstetric outcomes of women in different group

	OCP*(2010–2015) *N* = 18,415	TCP**(2016–2021) *N* = 20,601	F/t	*P*-value
**Maternal age (years)**	30.0 ± 4.46	32.3 ± 4.68		< 0.001
**AMA** ^**#**^	2687 (14.6%)	6502 (31.6%)	1555.247	< 0.001
**Multiparous**	4669 (25.4%)	9812 (47.6%)	2066.956	< 0.001
**Postpartum hemorrhage**	1016 (5.5%)	1217 (5.9%)	2.744	0.098
**Gestational age at delivery (weeks)**	38.6 ± 1.92	38.5 ± 1.82		0.059
**Vaginal delivery**	7270 (39.5%)	8923 (43.3%)	58.895	< 0.001
**Forceps**	1168 (6.3%)	1293(6.3%)	0.072	0.788
**Cesarean**	9885(53.7%)	10,281(49.9%)	55.451	< 0.001
**Stillbirth**	211 (1.1%)	150 (0.7%)	18.469	< 0.001
**Birth weight (g)**	3127 ± 511.9	3102 ± 493.1		< 0.001
**Newborn gender**				
**Male**	9786 (53.1%)	10,855(52.7%)	0.790	0.374
**Female**	8629 (46.9%)	9746 (47.3%)		
**Neonatal asphyxia**	626 (3.4%)	697 (3.4%)	0.001	0.979
**Neonatal death**	15 (0.1%)	5 (0.0%)	6.206	0.013
**Preterm birth**	1734 (9.4%)	2062 (10.0%)	3.893	0.048

*OCP: one-child policy; **TCP: two-child policy

#AMA: advanced maternal age, women age ≧ 35 years old

### Delivery mode and obstetric outcomes

**Fig. 2:**
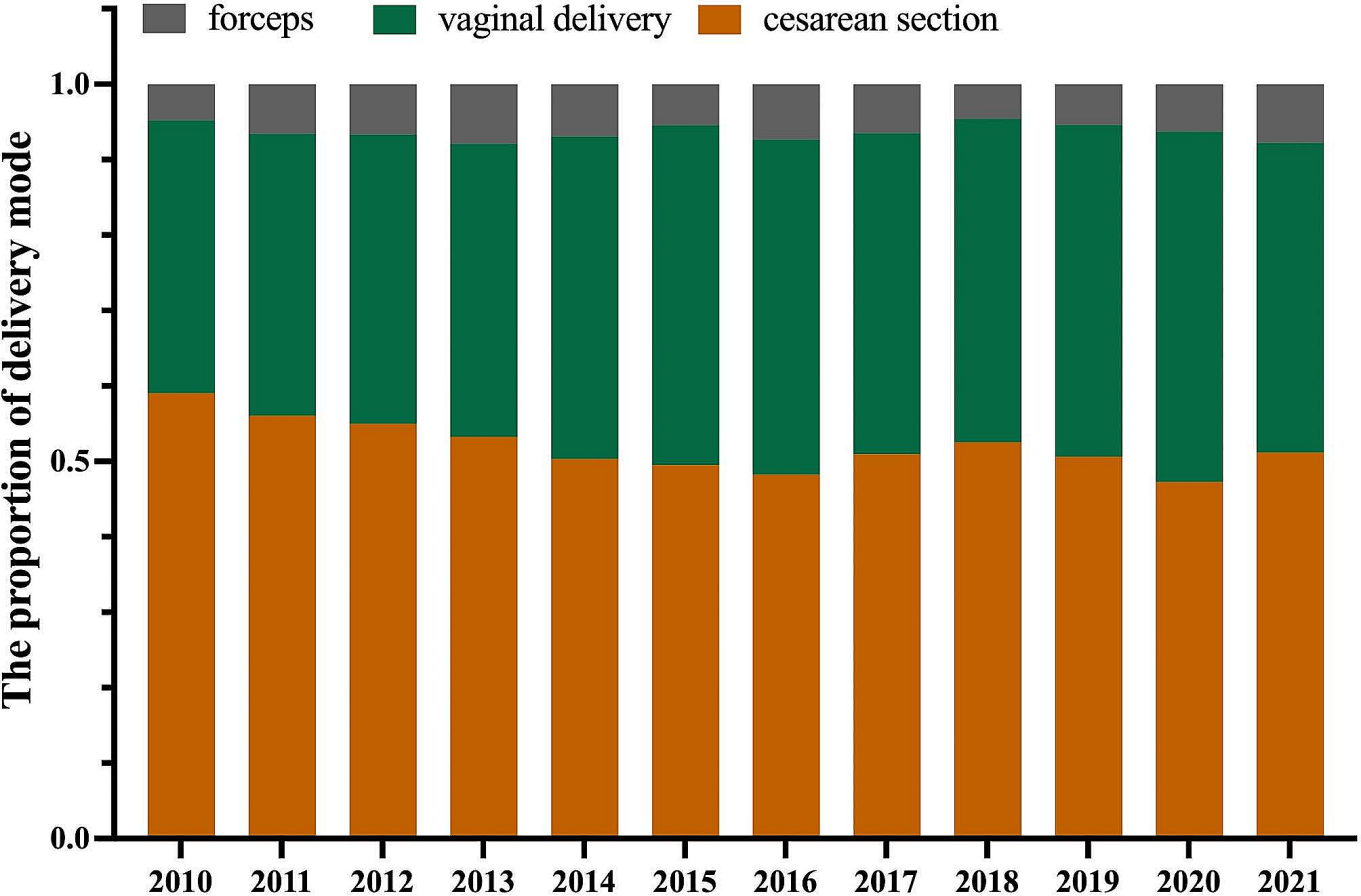
The delivery mode before and after universal child policy (from 2010 and 2021)

Table 2 shows the obstetric outcomes after the change in the family planning policy. As shown in Fig. 2, a dramatic drop in the rate of cesarean section was observed during the 5-year TCP (*P* < 0.001) period, while a 1.10-fold increase in the proportion of vaginal deliveries occurred after the family planning policy changed (*P* < 0.001). After adjusting for maternal age, parity and fetal distress, the rate of cesarean section still exhibited a decrease in the TCP period (RR: 0.745, 95%CI: 0.714–0.777, *P* < 0.001), suggesting that the universal two-child policy exerted a favorable influence on the rate of cesarean section. Furthermore, multiparity was associated with a lower rate of cesarean section(RR: 0.921, 95%CI: 0.882–0.962, *P* < 0.001), while AMA was associated with a higher rate of cesarean section (RR: 2.569, 95%CI: 2.442–2.703, *P* < 0.001).

**Table 2:** The demographic characteristics and obstetric outcomes of women with advanced maternal age

	OCP*(2010–2015) *N* = 18,415	TCP**(2016–2021) *N* = 20,601	F/t	*P*-value
**Maternal age (years)**	37 ± 4.5	38 ± 2.5		< 0.001
**Multiparous**	1136 (42.3%)	2829 (43.5%)	1.177	0.278
**Postpartum hemorrhage**	164 (6.1%)	348 (5.4%)	2.040	0.153
**Gestational age at delivery (weeks)**	38.1 ± 2.04	38.2 ± 1.77		0.002
**Vaginal delivery**	620 (23.1%)	1997 (30.7%)	54.478	< 0.001
**Forceps**	87 (3.2%)	237(3.5%)	0.927	0.336
**Cesarean**	1968(73.2%)	4246(65.3%)	54.728	< 0.001
**Stillbirth**	32 (1.2%)	27 (0.4%)	17.914	< 0.001
**Birth weight (g)**	3107 ± 541.1	3109 ± 500.5		0.871
**Newborn gender**				
**Male**	1442 (53.7%)	3385(52.1%)	1.964	0.161
**Female**	1245 (46.3%)	3117 (47.9%)		
**Neonatal asphyxia**	83 (3.4%)	214 (3.3%)	0.000	0.982
**Neonatal death**	15 (0.1%)	5 (0.0%)	6.206	0.013
**Preterm birth**	768 (11.8%)	331 (12.3%)	0.464	0.496
**Fetal distress**	69 (2.6%)	252 (3.9%)	9.646	0.002
**PROM** ^**#**^	134 (5.0%)	664 (10.2%)	65.461	< 0.001

*OCP: one-child policy; **TCP: two-child policy

#PROM: premature rupture of membrane

**Fig. 3:**
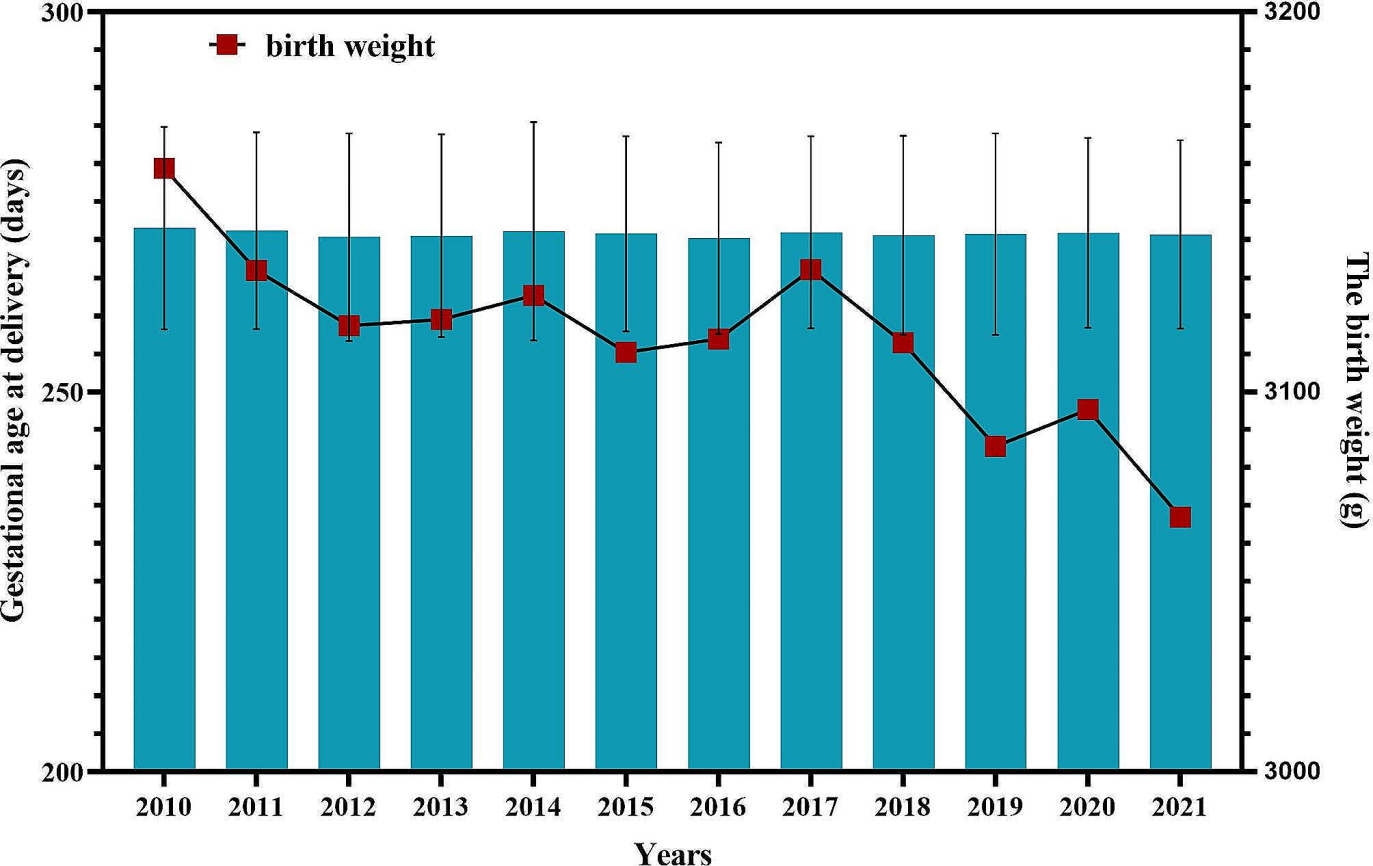
The trend of gestational age at delivery and birth weight before and after universal two-child policy (from 2010 and 2021)

Compared with that during the OCP period, the risk of postpartum hemorrhage during the TCP period increased to 5.9% (*P* = 0.098), but the change was not significant. There were 1016 cases suffered from PPH in OCP, including 423, 148 and 445 from vaginal delivery, forceps and cesarean section, respectively. A total 1217 PPH cases in TCP, containing 636, 218 and 363 from vaginal delivery, forceps and cesarean section, respectively. The proportion of preterm births increased from 9.4% in the OCP period to 10.0% in the TCP period (*P* = 0.048). Similarly, the risks of stillbirth and neonatal death significantly increased over time (*P* < 0.001). However, more pregnancies involved fetal distress in the TCP period. There was no significant difference in the gestational age at delivery, risk of neonatal asphyxia or the sex ratio of offspring between the two periods. The mean birth weights were 3127 ± 511.9 g and 3102 ± 493.1 g in the OCP period and the TCP period, respectively. A declining trend was in neonatal weight was observed from 2010 to 2021 (Fig. 3).

### Obstetric characteristics in different maternal ages

We categorized women into two groups according to their age at pregnancy. Table 3 shows that the rate of multiparity significantly increased from the OCP period to the TCP period among women under 35 years old (*P* < 0.001), as did the risk of postpartum hemorrhage (*P* = 0.006). Compared to the OCP period, the rate of cesarean section decreased from 50.3 to 49.1% in the TCP period (*P* < 0.001). There was no significant difference in the risks of preterm birth and neonatal asphyxia. The incidence of stillbirth (*P* = 0.022) and neonatal death (*P* = 0.001) decreased in the TCP period, whereas the risk of fetal distress increased (*P* < 0.001). The impact of the universal two-child policy on the rate of cesarean section and postpartum hemorrhage was investigated by multivariable-adjusted logistic regression analysis. After adjusting for parity and fetal distress (Table 4), we found a significantly negative association between the universal two-child policy and cesarean section (RR: 0.754, 95%CI: 0.718–0.790, *P* < 0.001). Furthermore, as shown in Table 5, the universal two-child policy increased the risk of postpartum hemorrhage (RR: 1.135; 95%CI: 1.025–1.257; *P* = 0.015); this risk was not mediated by parity (RR: 1.002; 95%CI: 0.901–1.115, *P* = 0.966) or fetal distress (RR: 1.471, 95%CI: 1.217–1.776, *P* < 0.001).

**Table 3:** The demographic characteristics and obstetric outcomes of women under 35 years old

	OCP*(2010–2015) *N* = 18,415	TCP**(2016–2021) *N* = 20,601	F/t	*P*-value
**Maternal age (years)**	29 ± 3.0	30 ± 2.9		< 0.001
**Multiparous**	3533 (22.5%)	6983 (49.5%)	2385.827	< 0.001
**Postpartum hemorrhage**	852 (5.4%)	869 (6.2%)	7.619	0.006
**Gestational age at delivery (weeks)**	38.7 ± 1.88	38.7 ± 1.82		0.237
**Vaginal delivery**	6650 (42.3%)	6926 (49.1%)	140.373	< 0.001
**Forceps**	1081 (6.9%)	1056(7.5%)	4.252	0.039
**Cesarean**	7917(50.3%)	6035(42.8%)	169.435	< 0.001
**Stillbirth**	179 (1.1%)	123 (0.9%)	5.220	0.022
**Birth weight (g)**	3130 ± 5406.7	3100 ± 489.7		< 0.001
**Newborn gender**				
**Male**	8342 (53.0%)	7383(47.0%)	0.015	0.904
**Female**	7469 (53.0%)	6629 (47.0%)		
**Neonatal asphyxia**	538 (3.4%)	483 (3.5%)	0.012	0.913
**Neonatal death**	15 (0.1%)	1 (0.0%)	10.807	0.001
**Preterm birth**	1403 (8.9%)	1294 (9.2%)	0.600	0.439
**Fetal distress**	665 (4.2%)	901 (6.4%)	69.882	< 0.001
**PROM** ^**#**^	849 (5.4%)	1834 (13.0%)	525.952	< 0.001

*OCP: one-child policy; **TCP: two-child policy

#PROM: premature rupture of membrane

**Table 4:** The multivariate logistic regression model for the risk of cesarean section

	All the women RR (95%CI)	*P*	AMA^#^ group RR (95%CI)	*P*	< 35 years old RR(95%CI)	*P*
TCP*	0.745 (0.714–0.777)	< 0.001	0.898 (0.628–0.767)	0.017	0.754 (0.718–0.790)	< 0.001
**AMA** ^**#**^	2.569 (2.442–2.703)	< 0.001	-	-	-	-
**Multiparous**	0.921 (0.882–0.962)	< 0.001	0.694 (0.822–0.981)	< 0.001	0.929 (0.884–0.977)	0.004
**Fetal distress**	0.870 (0.791–0.956)	0.004	0.499 (0.399–0.625)	< 0.001	0.975 (0.880–1.081)	0.634

*TCP: two-child policy

#AMA: advanced maternal age

**Table 5:** The multivariate logistic regression model for the risk of postpartum hemorrhage

	AMA group RR (95%CI)	*P*	< 35 years old RR(95%CI)	*P*
TCP*	0.864(0.713–1.046)	0.194	1.135(1.025–1.257)	0.015
**Multiparous**	1.234(1.032–1.475)	0.021	1.002 (0.901–1.115)	0.966
**Fetal distress**	1.334(0.864–2.059)	0.194	1.471 (1.217–1.776)	< 0.001

*TCP: two-child policy

Among pregnant women 35 years of age or older (Table 2), 65.3% of pregnancies underwent cesarean section in the TCP period, which steadily decreased after the two-child policy was implemented compared with that in the OCP period (73.2%, *P* < 0.001). The significance of this association remained (RR: 0.898; 95%CI: 0.628–0.767; *P* = 0.017) after adjusting for parity (RR: 0.694; 95%CI: 0.822–0.981; *P* < 0.001) and fetal distress (RR: 0.499; 95%CI: 0.399–0.625; *P* < 0.001) were added to the adjustment (Table 4). Compared to women in the OCP period, in the TCP period only 0.4% experienced stillbirth (*P* < 0.001), and 5 cases resulted in neonatal death (*P* < 0.001). As shown in Table 2, there was no significant change in postpartum hemorrhage, preterm birth or parity between the two periods. Stratified analysis confirmed that the association between the universal two-child policy and postpartum hemorrhage was stronger in young women and disappeared in women with AMA (Table 5).

## Discussion

In China, the one-child policy lasted for more than 30 years, producing health consequences such as low birth rate, population aging, and a higher rate of cesarean Sect. [2]. In this study, we retrospectively explored changes in maternal characteristics and obstetric outcomes in patients at one tertiary center over 10 years (from 2010 to 2021), an interval centered around the implementation of the universal two-child policy in 2016. Following the introduction of the universal two-child policy, we found an increased number of pregnancies in multiparous women or women with AMA. Furthermore, the rate of cesarean section decreased after the universal two-child policy was implemented, regardless of maternal age. After adjusting for potential confounders, including maternal age, parity and fetal distress, the risk of cesarean section still decreased after the introduction of the universal two-child policy. Nevertheless, fetal distress and neonatal asphyxia were observed more frequently observed during 5-year TCP period. Although a trend of increased risk of postpartum hemorrhage was detected during the TCP period, the implementation of the universal two-child policy increased the risk of postpartum hemorrhage in pregnant women under 35 years of age.

Interestingly, the number of pregnancies exhibited a rapid increase during the first two years of the TCP period and then rebounded slightly. Since the universal two-child policy overall had a positive impact on the number of pregnancies, we speculate that its influence on the desire for a second child desire was mild. Other explanations for the decreased birth rate between 2019 and 2021 include delays in childbearing, reproductive health and the high economic cost of raising children [7]. The desire for children is affected by a couple’s socioeconomic status in addition to the healthcare system [8]. Chang found that financial resources to childbearing and additional support would affect the decision to have another child [9].

In the present study, a sharp increase in the number of pregnant women over 35 years old was observed after the introduction of the universal two-child policy. Although delayed childbearing is observed across the globe, the universal two-child policy might have encouraged older women to want a second child. In line with our findings, Li [10] identified that pregnancies with AMA increased 5.8% each month between 2016 (the start of the TCP period) and 2017. In addition, Chinese national surveillance data indicated that the rate of pregnant women with AMA increased from 7.8 to 10.9% over this period [11]. Based on this evidence [12], AMA seems to be correlated with obstetric complications, including gestational diabetes mellitus, hypertension disorder of pregnancy, placental abruption and preterm birth. Zhang [13] found that the number of hypertension, placenta previa and postpartum hemorrhage cases significantly increased during the period of the TCP period. However, we also investigated whether the proportion of fetal distress, stillbirth and neonatal death cases declined during the TCP period in women with AMA. There was no significant difference in the rate of postpartum hemorrhage among women with AMA after the announcement of the universal two-child policy. This improvement in obstetric outcomes, regardless of AMA, might be attributed to governmental investment in pregnancy health care services [14].

In present study, most cases admitted in our hospital were high-risk pregnancies, leading to high rate of cesarean section. After the universal two-child policy was introduced, the rate of cesarean section slightly decreased from 53.7 to 49.9%, particularly in nulliparous women, however, it remained relatively high. One plausible explanation for this decrease was the implementation of the universal two-child policy and comprehensive interventions to reduce unnecessary cesarean Sect. [15]. Another potential cause was the preference of nulliparous women for vaginal delivery to prevent the potential adverse consequence of cesarean section in the following pregnancy [16]. The implementation of painless childbirth, promotion of health care, and encouragement of vaginal delivery as well as financial incentives have changed decisions regarding delivery mode, leading to a drop in cesarean Sect. [1]. Notably, the percentage of cesarean section was still increased in women over 35 years old [17], consistent with a previous study. Because our hospital is the best hospital in South China, more than 90% of cases were high-risk pregnancies. Severe complications were responsible for the high cesarean section rate. Although previous cesarean section was the major influence on delivery mode in pregnancies among older women [16], we found that 43.3% of pregnancies resulted in vaginal delivery after the implementation of the universal two-child policy. The Chinese government has also introduced strategies, including renewing clinical guidelines for cesarean section indications and training midwives to reduce the number of unnecessary cesarean Sect. [18]. The increased quality of maternal care might also have promoted vaginal delivery in the TCP period. However, these causes are speculative and not necessarily due to the shift in population policy. Thus, caution is warranted when interpreting the decrease in the cesarean section rate following the change in family planning policies. Nevertheless, the negative association between the universal two-child policy and the risk of cesarean section in our study is biologically plausible.

Recently, a significantly increased risk of preterm birth was reported in the TCP period. Zhang [19] observed that more pregnant women who delivered before 37 gestational weeks suffered from complications, including preeclampsia, oligohydramnios and placenta previa, during the TCP period. In contrast to previous studies [14], we found no significant difference in the risk of preterm birth in pregnancies among women with AMA during the TCP period. In our subgroup analysis, the risk of preterm birth was similar in pregnant women under 35 years old before and after the introduction of the universal two-child policy.

We also found that there was an increasing trend in the risk of postpartum hemorrhage after the introduction of the universal two-child policy. In accordance with previous studies, AMA, prior cesarean section and placenta previa were associated with postpartum hemorrhage [20]. A plausible cause is that in the TCP period, more pregnancies with AMA face a higher risk of complications with hypertension, gestational diabetes mellitus, thyroid disease, anemia and so on [21]. Interestingly, after the introduction of the universal two-child policy, there was no significant difference in the risk of postpartum hemorrhage among women with AMA, while pregnant women under 35 years of age were more likely to suffer from postpartum hemorrhage. After adjusting for parity and fetal distress, the implementation of the universal two-child policy was still positively correlated with rates of postpartum hemorrhage in younger women.

It has been demonstrated that advanced maternal age is associated with adverse neonatal outcomes [22]. However, in our study, the incidence of stillbirth and neonatal death significantly decreased after the introduction of the universal two-child policy despite the increases in maternal age. This discrepancy might be attributed to the more comprehensive maternity and neonatal care network. The gestational age at birth and mean neonatal birth weight were similar before and after implementation of the universal two-child policy, indicating that infant health status was similar. Owing to improvements in pregnancy management, such as increased health evaluations, antenatal visits and nutritional guidance, neonatal outcomes have improved after implementing the universal two-child policy [10]. Although the universal two-child policy did not impact the neonatal outcomes, we found that the rate of fetal distress increased in the TCP period. A possible cause was that the pregnancies with AMA increased in the TCP period, leading to the incidence of fetal distress.

Although the risk of cesarean section decreased in the TCP period, it was more common in women with AMA. The lack of reduction in this age group might be explained by the risk of obstetric complications, which are more likely in pregnancies of women with AMA, leading to a heightened risk of cesarean Sect. ^[9]^. After the implementation of the universal two-child policy, more women who underwent uterine surgery became pregnant [23] or utilized IVF [24], resulting in cesarean delivery. The number of pregnancies complicated with severe disease increased during the TCP period, which played an important role in the rate of cesarean section among the women with AMA over the study period. Moreover, the risk of preterm birth also increased in the TCP period, owing to complications. Perinatal care should be improved for women with AMA, particularly in the period of new population policy.

In this study, we found that postpartum hemorrhage was common in women under 35 years old in TCP. Joep [25] found that the risk of postpartum hemorrhage increased in nulliparous women with AMA. In our cohort, more nulliparous women were detected in women under 35 years old in TCP. In a retrospective case control study [26], containing 34 175 pregnancies, prolonged labor was identified as a risk factor for severe postpartum hemorrhage. Among pregnancies under 35 years old, prolonged labor might be frequently observed in TCP due to parity. We did not have a certain explanation for this controversial finding. The proportion of high-risk pregnancies under 35 years old increased slightly during the TCP period, leading to intractability and management challenges. Therefore, we need to identify the risk factors for postpartum hemorrhage during antenatal care and provide early intervention to prevent postpartum hemorrhage in women under 35 years old during the period of new population policy.

A strength of this paper was its large sample size over the past decade, allowing a thorough investigation of the changes accompanying the implementation of the universal two-child policy in China. The findings provide unique insights into the delivery mode and the changes in obstetric characteristics after the change in family planning policies. Nevertheless, some limitations in terms of analysis remain. First, this study was retrospective and focused on data from one tertiary hospital in southern China; thus, there is potential selection bias. Most of cases were high-risk pregnancies in this study, leading to data bias. The proportion of high-risk pregnancies was various in different regions of China. Different demographic characteristic distributions and determinants among Chinese women with singleton pregnancy can make the results less generalizable to other regions and countries. Second, we excluded twin and multiple pregnancies, which might limit the generalizability of our findings. Third, numerous pregnancies complicated with severe disease were transferred to our hospital, leading to a relatively high rate of cesarean section. The number of cases with some complications did not meet the requirement of statistical analysis. Furthermore a larger size of cohort should be investigated to address new challenges related to the universal 2-child policy in China.

## Conclusions

In summary, after implementation of the universal two-child policy, more pregnancies occurred in women with AMA, and women were more likely to have multiple children. The rate of cesarean section significantly declined in the TCP period after adjusting for parity and fetal distress. After adjusting for the same confounding factors, the risk of postpartum hemorrhage in the TCP period was similar in pregnant women ≥ 35 years old, but increased in pregnant women < 35 years old. As the Chinese government has announced a three-child policy, the findings of this study highlight the increasing trend of AMA among pregnant women for obstetricians as well as adverse obstetric outcomes.

## Data Availability

The datasets generated and/or analyzed during the current study are not publicly available due to patient privacy but are available from the corresponding author on reasonable request.
